# Exploring the use of manual therapy in the management of traumatic brain injury: a scoping review

**DOI:** 10.1186/s12998-025-00606-y

**Published:** 2025-10-10

**Authors:** Tristan Delion, Aurelien Noyer, Matthieu Gonzalès-Bandrès, Loïc Treffel, Gerard Farrell, Hélène Cassoudesalle, Mathieu Ménard

**Affiliations:** 1 Present Address: Collège Ostéopathique de Bordeaux, 33700 Mérignac, France; 2 The UCO School of Osteopathy, Health Science University, London, UK; 3 Institut Dauphine d’Ostéopathie, 75005 Paris, France; 4 Institut Toulousain d’Ostéopathie, Pôle Recherche, 31670 Toulouse, France; 5 Faculty of Public Health, UTS-ARCCIM, Sydney, Australia; 6 Centre for Health, Activity, and Rehabilitation Research, School of Physiotherapy, University of Otago, Dunedin, New Zealand; 7 Université de Bordeaux, INSERM, BPH, U1219, ACTIVE Team, Bordeaux, France; 8 Université Rennes, IO-RB, INRIA, M2S, Rennes, France

**Keywords:** Traumatic brain injury, Post-concussion syndrome, Musculoskeletal manipulations, Chiropractic, Osteopathy, Physiotherapy

## Abstract

**Objectives:**

To comprehensively map the literature on the use of manual therapy (MT) in the management of traumatic brain injury (TBI).

**Background:**

TBI is a leading cause of long-term disability worldwide, often resulting in persistent physical, cognitive, and emotional symptoms. MT, which is commonly used by chiropractors, osteopaths and physiotherapists (COPs), has been proposed as a nonpharmacological intervention for post-TBI symptom management. However, the scope of supporting evidence remains unclear.

**Design:**

Scoping Review.

**Methods:**

Four databases and Google Scholar were searched for peer-reviewed studies published in English or French from 2010 onwards. The inclusion criteria targeted all severities of TBI, with MT interventions delivered by COPs. Data extraction and assessment of methodological reporting were conducted independently by two reviewers via standardised tools.

**Results:**

Forty-two articles were included, comprising primarily case reports, case series, and randomised controlled trials. Most studies have investigated mild, sport-related TBI and described MT interventions targeting headache, neck pain, and dizziness—often delivered within multimodal care frameworks. A subset of studies has also explored the impact of MT on cognitive, emotional, or sleep-related symptoms, suggesting potential benefits beyond physical outcomes. Moderate to severe TBI has rarely been examined. Reporting limitations were common, particularly regarding adverse events.

**Conclusion:**

The literature reflects a growing interest in the use of MT for mild, sport related TBI, primarily for managing physical symptoms. Further research is needed to explore broader applications across different populations and TBI severities, investigate underlying MT mechanisms and improve the reporting of safety outcomes.

**Supplementary Information:**

The online version contains supplementary material available at 10.1186/s12998-025-00606-y.

## Introduction

Traumatic brain injury (TBI) is estimated to affect 50 to 60 million people worldwide annually and imposes a significant global economic burden of approximately 400 billion US dollars per year [1]. TBI can affect individuals across their lifespan, from early childhood to older adulthood [2]. The Mayo Classification System provides a framework for determining injury severity, distinguishing three categories: moderate-severe, mild and symptomatic [3]. The latter two are often grouped together under the broader category of mild TBI, which accounts for approximately 81% of cases [4]. However, irrespective of severity, all forms of TBI can lead to long-term disability [5, 6] characterised by persistent cognitive, psychological and physical symptoms [7].

Mild TBI is defined as traumatic physiological disruption of brain function resulting from external mechanical forces [8]. Although the term ‘concussion’ has been criticised for lacking pathological precision [9], recent consensus supports its interchangeable use with mild TBI when neuroimaging is normal or not clinically indicated [8]. To address the challenges in diagnosing mild TBI, the American Congress of Rehabilitation Medicine (ACRM) has recently developed new diagnostic criteria [8]. This updated approach provides a standardised case definition by specifying the signs, symptoms, and examination findings required for diagnosis, and thereby improving consistency across research studies and clinical settings. This clarification is important in the context of emerging research aimed at developing guidelines to support the recognition and management of mild TBI [10].

Recent systematic reviews have investigated the effectiveness of nonpharmacological interventions for managing mild TBI symptoms such as headache, neck pain, dizziness and cognitive difficulties [11–13]. Favourable outcomes were reported for tailored, multimodal interventions, including neuropsychology, occupational therapy, physiotherapy, and vestibular and oculomotor therapy. Among these interventions, manual therapy (MT) targeting the cervical spine has shown promising results in managing neck pain in individuals with concussion [11–13]. However, both the consensus statements and systematic reviews emphasise the need for further research to clarify the role of rehabilitation strategies, including MT, within interdisciplinary care models [10–13].

The management of moderate to severe TBI in acute care typically involves a complex multidisciplinary approach aimed at stabilising vital functions, preventing secondary injury and initiating early rehabilitation to optimise neurological recovery and functional outcomes [14]. Despite growing interest in physiotherapy during the subacute and chronic phases of recovery, evidence remains limited regarding the specific contribution of MT within this context [15].

MT can be defined as a patient-centred, hands-on approach involving the application of mechanical force to the body with therapeutic intent. It includes a wide range of techniques, such as light touch, soft tissue techniques, and thrust and non-thrust manipulations, and is commonly used across various physical therapy disciplines to address pain, support rehabilitation and promote overall health [16]. Among these professions, chiropractors, osteopaths and physiotherapists (COPs) frequently incorporate MT into their practice [17]. Recent ethnographic and qualitative research has revealed the intersectionality and overlapping clinical practices of COPs in the management of musculoskeletal disorders, where MT is frequently integrated into practice [18, 19]. Growing evidence supports the use of MT for symptoms frequently experienced after TBI, including neck pain [20, 21], headaches [22] and dizziness [23], but its implementation in broader interdisciplinary rehabilitation frameworks for TBI remains unclear.

Despite the emergence of recent studies exploring the use of MT as part of nonpharmacological care for individuals with TBI, the scope, quality, and consistency of the evidence remain limited and heterogeneous. Therefore, a scoping review was deemed appropriate to comprehensively map the literature, identify current trends, explore methodological characteristics and reporting, and highlight gaps in MT research for individuals with TBI.

## Methods

This scoping review was conducted in accordance with the framework proposed by Arskey and O’Malley in 2005 [24] and the Joanna Brigg Institute recommendations for scoping reviews [25] and is reported following the Preferred Systematic Reviews and Meta-Analysis Protocols, Scoping Reviews extension (PRISMA-ScR) guidelines [26]. The objectives, inclusion criteria and methods were specified in advance and documented in an a priori protocol registered on the Open Science Framework: 10.17605/OSF.IO/GQRUF.

### Inclusion criteria

#### Participants

The review considered studies including participants diagnosed with any severity of TBI, covering all related symptoms and injury phases, with no restrictions on age or sex. Studies involving acquired brain injury other than TBI and animal studies were excluded.

#### Concept

MT interventions delivered by COPs, either as standalone interventions or as part of complex interventions, were included. Studies were excluded if MT was not delivered by COPs.

#### Context

Studies conducted in countries where COP is practiced were included. Studies not published in French or English were excluded.

The detailed inclusion and exclusion criteria are presented in Table 1.

**Table 1:** Eligibility criteria

Domain	Inclusion criteria	Exclusion criteria
Population	Studies involving individuals diagnosed with any severity of TBI, with no restriction on age, sex or injury phase	Studies involving animal models, participants without a diagnosis of TBI, or with acquired brain injury not specifically defined as TBI (e.g.; stroke). Studies focused solely on whiplash were also excluded
Intervention/exposure	MT (including light touch, massage, joint mobilisation or manipulation), delivered by COPs. Studies were included if MT was part of a multidisciplinary or interdisciplinary approach, as long as MT was clearly defined as a component of the intervention	Studies in which MT was not described as part of the intervention, or where it was not delivered by COPs. Interventions using dry needling, acupuncture, or instrument-assisted techniques were excluded
Outcomes	Any outcomes related to TBI symptomatology including physical, cognitive, emotional and sleep-related symptoms. Validated measures such as the Rivermead Post-Concussion Questionnaires (RPQ) were accepted	No specific outcome-related exclusion criteria were applied
Study characteristics	Peer-reviewed empirical studies using either observational designs (e.g., descriptive, cohort, cross-sectional, or case–control) or experimental designs (e.g., randomised controlled trials, non-randomised trials, and quasi-experimental approaches such as pre–post studies) were included	Reviews, commentaries, guidelines, and studies not published in English or French or not peer-reviewed were excluded

*TBI* traumatic brain injury, *MT* manual therapy, *MSK* musculoskeletal, *COPs* chiropractors osteopaths physiotherapists

### Review questions

On the basis of the Population, Concept, Context (PCC) framework developed by Peters et al. in 2020 [25], the following review questions were proposed:

- What is the extent and nature of the existing evidence regarding MT intervention in the management of TBI?
- Which severities of TBI have been studied, and at what phases of the injury are interventions applied? What symptoms and outcomes are assessed, and which population is targeted?
- What types of MT are used, and in what context? Are any adverse events associated with MT interventions reported?
- What methodological features and reporting practices are observed in studies on MT in the context of TBI?

### Types of studies

This scoping review considered observational studies (descriptive studies, cohort studies, cross-sectional studies, and case‒control studies) and experimental studies (randomised controlled trials (RCTs), nonrandomised controlled trials, and quasi experimental designs such as before-after studies). We excluded grey literature to focus exclusively on peer-reviewed literature. To capture the most current evidence, only studies published from 2010 onwards were sought.

### Search strategy

The search strategy followed the three-step methodology recommended by the Joanna Briggs Institute (JBI) [27]. First, an initial search was conducted on PubMed to identify relevant keywords. These terms, which appeared in titles, abstracts and subject headings, were used to construct the full search strategy. This step was led by the primary reviewer (TD) and refined with input from the wider research team (MM, GF, HC) with expertise in scoping review methodologies and/or in the fields of TBI and MT. Next, the full-search strategy was adapted to PubMed (Additional File 1), MEDLINE (EBSCO), PEDro and the Index to the Chiropractic Literature database [28–31]. Additional hand searches were performed on Google Scholar to ensure comprehensive coverage of the literature, particularly focusing on newly published studies. All the databases were searched from inception to July 6, 2024.

### Selection of evidence sources

All identified records were uploaded to Zotero (version 6.0.36), where duplicates were removed. The remaining studies were imported into Covidence (Veritas Health Innovation, Melbourne, Australia) to assist with screening and further duplicate removal. Two reviewers independently screened the titles and abstracts as part of a first screening selection, followed by a second round of full texts screening. In cases of disagreement, reviewers engaged in an argumentative discussion; unresolved conflicts were arbitrated by a third reviewer. The overall process was documented, including reasons for full-text exclusions, and is presented in the Results section.

### Data extraction

Relevant data were extracted via a charting table developed to align with the review objectives and questions [27]. Two reviewers (TD and MM) piloted the data extracted from three studies to ensure consistency. Data extraction was conducted independently and in duplicate. The lead reviewer (TD) used AI technology (ChatGPT-4, 4o, OpenAI, 2024) to assist in the data extraction process. Specifically, a standardised data charting template and full-text articles were uploaded into the AI interface, and ChatGPT was prompted to generate structured outputs. Each data point generated by ChatGPT was then carefully cross-checked against the full text article by the lead reviewer to ensure accuracy, resolve omissions, and correct any misinterpretations. The final extracted dataset was reviewed against a second extraction conducted independently by a second reviewer (MM, LT, AN, MG). This approach aligns with recent findings by Motzfeldt Jensen et al., who showed that ChatGPT can support accurate and reproducible data extraction when used with human oversight [32]. The extracted data included (1) study characteristics (authors, publication year, study aim and design), (2) population characteristics (sample size, demographics), TBI severity, injury phases and post-TBI symptoms, (3) context (country and profession), and (4) concepts—manual therapy intervention, outcome measures, and adverse events.

### Data synthesis and reporting of the results

The data were analysed via a descriptive approach to address the review questions. The results are presented narratively and are supported by tables and figures to enhance clarity and comprehensiveness. Data related to symptoms, outcome measures, population characteristics, MT interventions, body regions treated and combined interventions were coded and cleaned collaboratively between two reviewers (TD and AN) to ensure consistency and improve readability. All members of the research team reviewed and approved the final presentation of the results.

### Assessment of methodological characteristics:

A descriptive appraisal of the methodological characteristics and reporting practices of the included studies was performed independently and in duplicate. Reviewers (TD, MM, LT, AN, and MG) used the appropriate Joanna Briggs Institute checklists tailored to each study design [33]. The purpose of the appraisal was to explore how studies were designed and reported, and to identify common methodological limitations or gaps in the literature.

## Results

### Study inclusion criteria

**Fig. 1:**
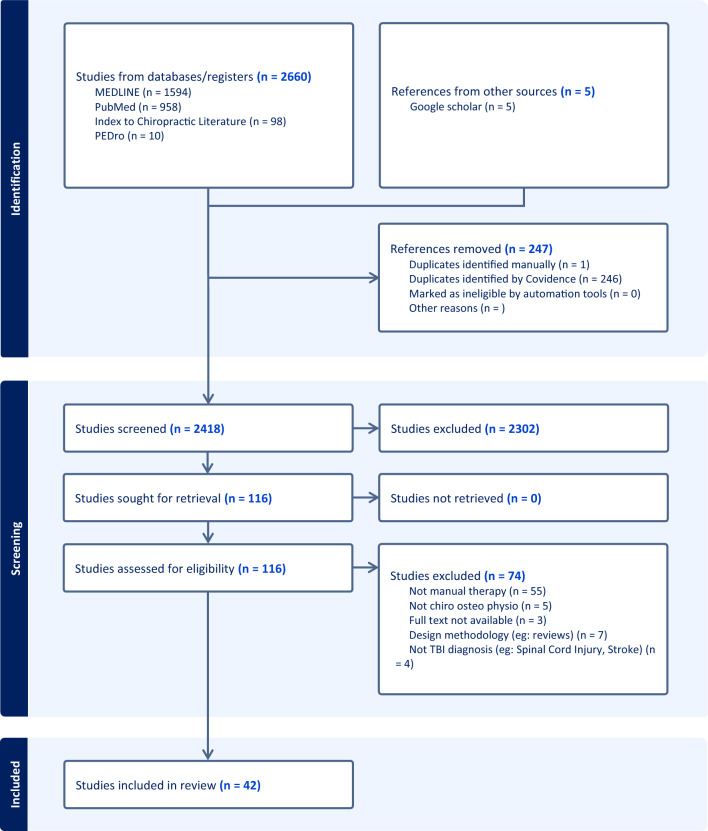
PRISMA Flow Chart

The selection process for sources of evidence is illustrated in the PRISMA-ScR flow chart (Fig. 1). A total of 2665 records were identified through database searches and manual searches via Google Scholar. After the removal of 247 duplicates, 2418 records were screened based on titles and abstracts. Among the 116 full-text articles assessed for eligibility, 42 met the inclusion criteria and were included in the final analysis. Independent reviewers demonstrated a substantial level of agreement during full-text screening (Cohen’s kappa = 0.80, 95% CI [0.10–1.50]) [34]. The reasons for exclusion at the full-text screening stage are presented in Fig. 1.

### Characteristics of the included studies

An overview of the studies’ characteristics is provided in Additional File 2.

### Article type

The most frequently represented study design was case reports (n = 19) [35–53], followed by RCTs (n = 10) [54–63], case series (n = 9) [64–72], experimental studies (n = 2) [73, 74], and cohort studies (n = 2) [75, 76].

### Participants and Practitioners

A total of 777 participants were included across the 42 studies. MT interventions were most frequently delivered by chiropractors (n = 18), followed by physiotherapists (n = 15) and osteopaths (n = 9).

### Research settings

Interventions were administered in a range of clinical and academic settings, including private clinics (n = 21), hospitals (n = 10), concussion specialist clinics (n = 5) and educational institutions (n = 10). Four studies reported interventions delivered across multiple settings [72, 74, 76].

### Review findings

#### Population characteristics

**Fig. 2:**
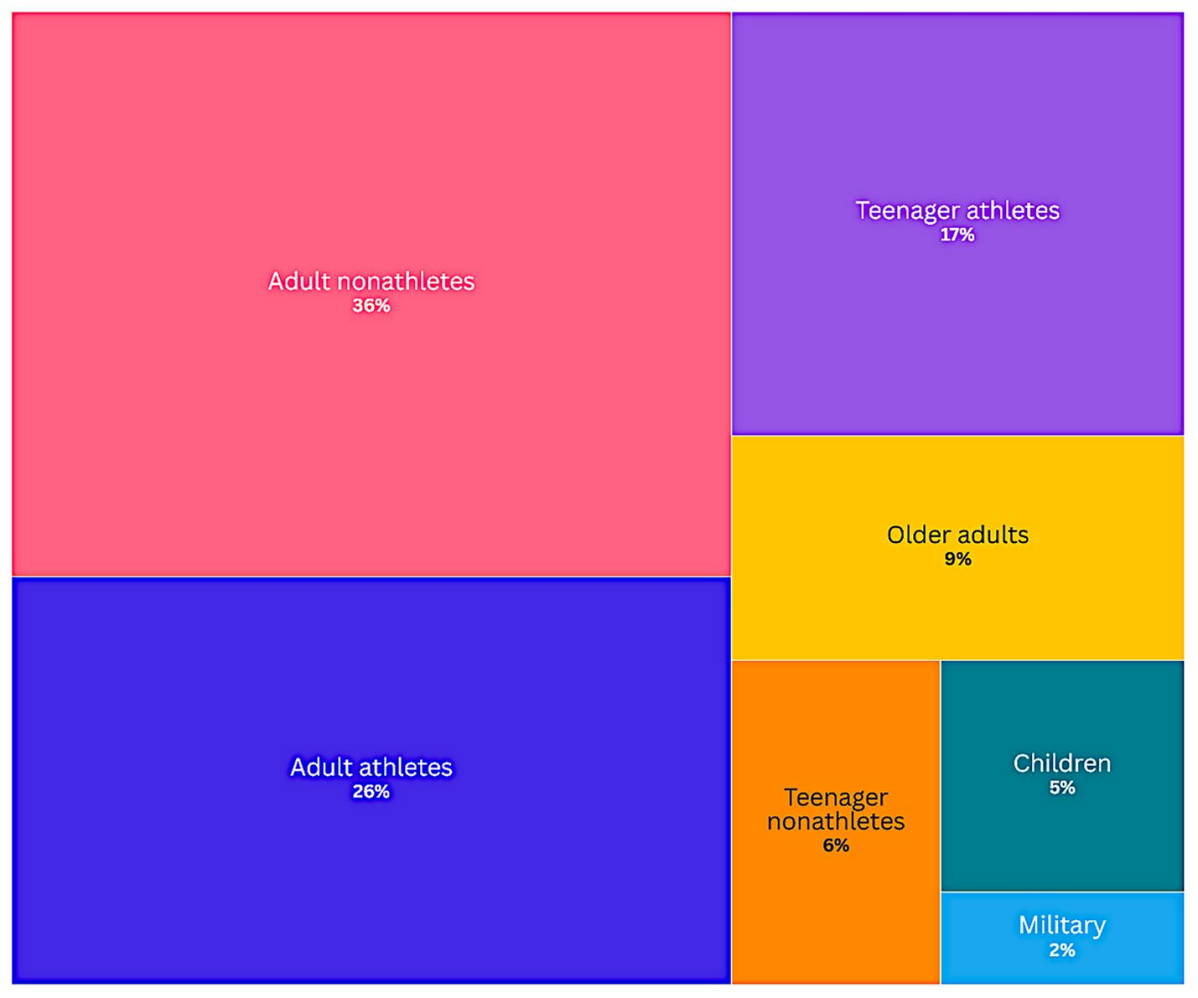
Studied population in percentages

The populations included in the selected studies were categorised into seven distinct groups, as shown in Fig. 2. Representations across these categories were unevenly distributed. Adult nonathletes constituted the largest proportion (36%), followed by adult athletes (26%) and teenage athletes (17%). In contrast, underrepresented groups included older adults (9%), teenage nonathletes (6%), children (5%) and military personnel (2%).

### TBI severity and injury phases

Most studies on manual therapy interventions were conducted in the context of mild TBI, with 37 of the 42 included studies targeting this population. Six studies addressed moderate to severe TBI, including one study [65] that investigated interventions across all TBI severity levels. Among the mild TBI cases, interventions were most frequently applied during the subacute (n = 23) and long-term phases (n = 22), with only four studies reporting interventions during the acute phase. Similarly, for moderate to severe TBI, interventions were primarily delivered in the subacute phase (n = 4), with the acute and long-term phases each represented only once. In one study, the timing of the intervention was not clearly reported [73]. The results are illustrated in Table 2.

**Table 2:** Timing of MT intervention across TBI severity and injury phases

TBI Severity	Injury Phase			
	Acute	Sub-Acute	Long Term	Unclear
Mild (n = 37)	4	23	22	0
Moderate-Severe (n = 6)	1	4	1	1

Numbers indicate the number of included studies reporting MT interventions at each injury phase. Some studies investigated interventions across multiple injury phases; therefore, counts per phase do not sum to the total number of studies within each severity group

*MT* Manual Therapy, *TBI* Traumatic Brain Injury

### Symptoms presentation

**Fig. 3:**
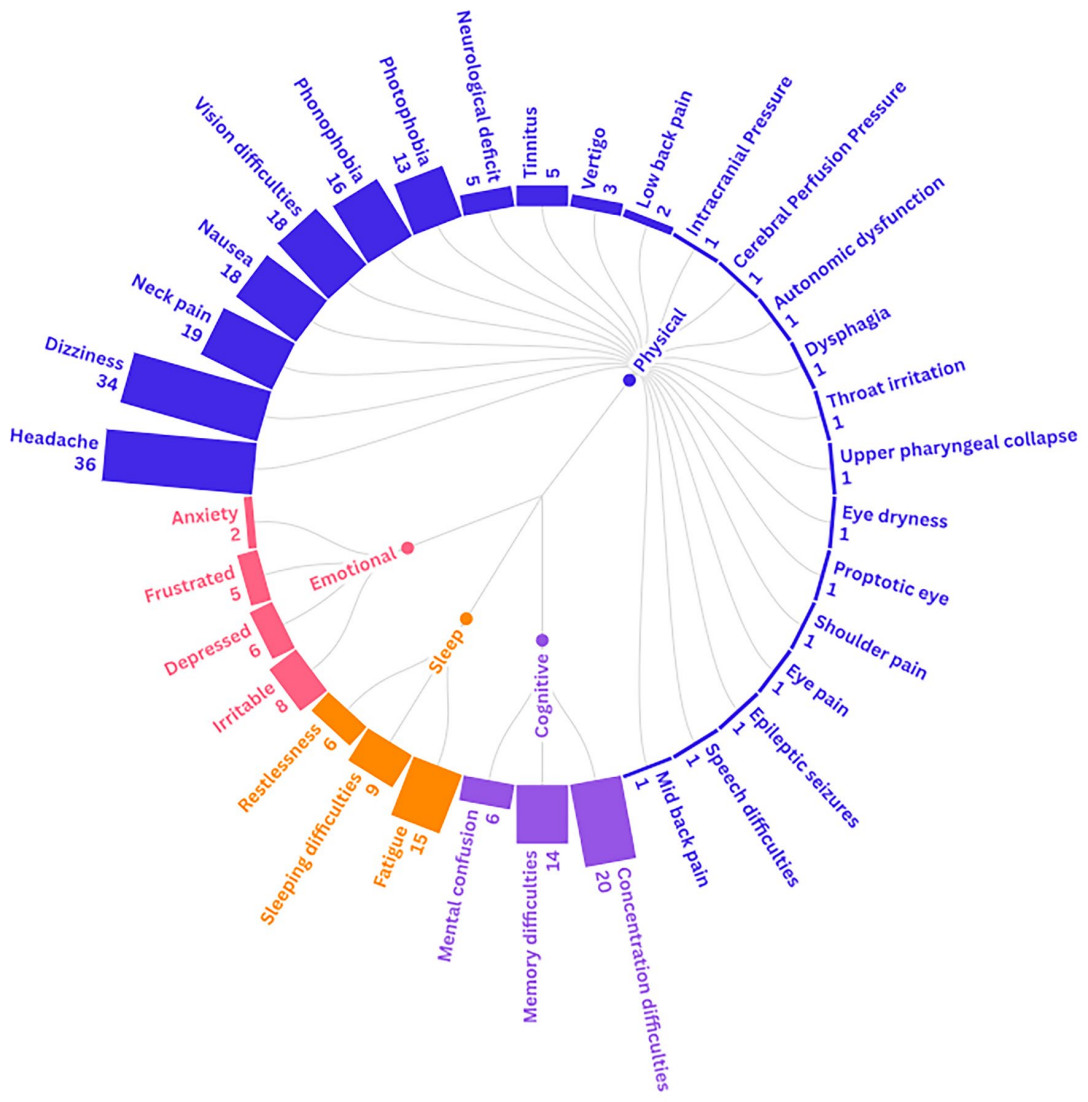
Frequency of symptom presentations across the included studies, classified by domain

Symptoms were categorised into four domains: physical, cognitive, sleep, and emotional. Within the physical domain, the most frequently reported symptoms were headache (n = 36), dizziness (n = 34), neck pain (n = 19), nausea (n = 18), visual disturbances (n = 18), phonophobia (n = 16), and photophobia (n = 13). In the cognitive domain, the most frequently reported symptoms were concentration difficulties (n = 20) and memory difficulties (n = 14). Emotional symptoms most frequently included irritability (n = 8) and depressive symptoms (n = 6). Within the sleep domain, fatigue (n = 15) was the most prevalent symptom. A full overview of symptoms identified across the included studies is provided in Fig. 3.

### Outcome measures used to assess MT interventions

**Fig. 4:**
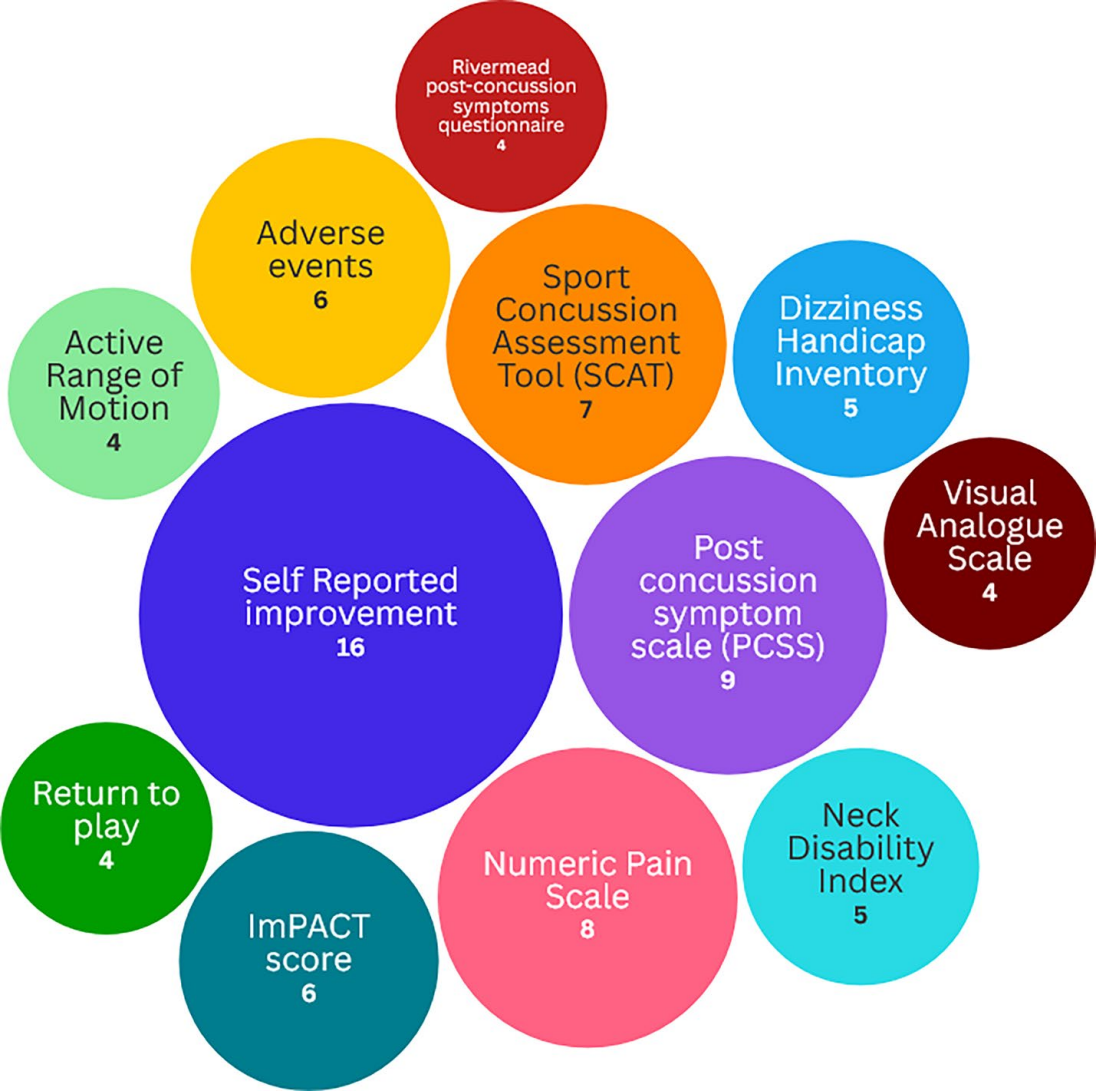
Frequencies of the most reported outcome measures in the included studies. Abbreviations used: ImPACT = Immediate post-concussion assessment and cognitive testing

The outcome measures used to evaluate the role of MT in the management of TBI were highly heterogeneous. A range of subjective assessment tools was reported, including self-reported improvement (n = 19), the Numeric Pain Scale (NPS) (n = 8), and the visual analogue scale (VAS) (n = 4). Standardised outcome measures, such as the Post-Concussion Symptom Scale (PCSS) (n = 9), the Immediate Post-Concussion Symptoms Assessment and Cognitive Testing (ImPACT) score (n = 6), the Dizziness Handicap Inventory (DHI) (n = 5), the Neck Disability Index (NDI) (n = 5), the Rivermead Post-Concussion Symptoms Questionnaire (RPQ) (n = 4), and the Headache Disability Inventory (HDI) (n = 3), were also frequently used. The Sport Concussion Assessment Tool (SCAT), which incorporates the PCSS, was used in seven studies. Biological markers, such as salivary cortisol and heart rate variability (HRV) were assessed in one study [56]. The full list of outcome measures can be found in Additional File 2, while Fig. 4 illustrates the most frequently used measures.

### Manual therapy interventions, body regions treated and professions

Manual therapy techniques were categorised into six groups. The most frequently reported methods were thrust manipulation (n = 23), non-thrust manipulation (n = 18), and soft tissue techniques (n = 18). Less commonly described approaches include functional techniques (n = 10), muscle energy techniques (MET) (n = 4), and neurodynamic techniques (n = 1). In four studies, the manual therapy techniques used were not specified [68, 69, 71, 76].

Interventions were applied to a range of body regions, with the cervical (n = 32) and thoracic (n = 22) areas being the most frequently treated. Less commonly treated regions included the lumbar spine (n = 9), pelvis (n = 9), head (n = 8), and lower extremities (n = 2). In four studies, the specific body regions treated were not specified [39, 59, 64, 65].

**Fig. 5:**
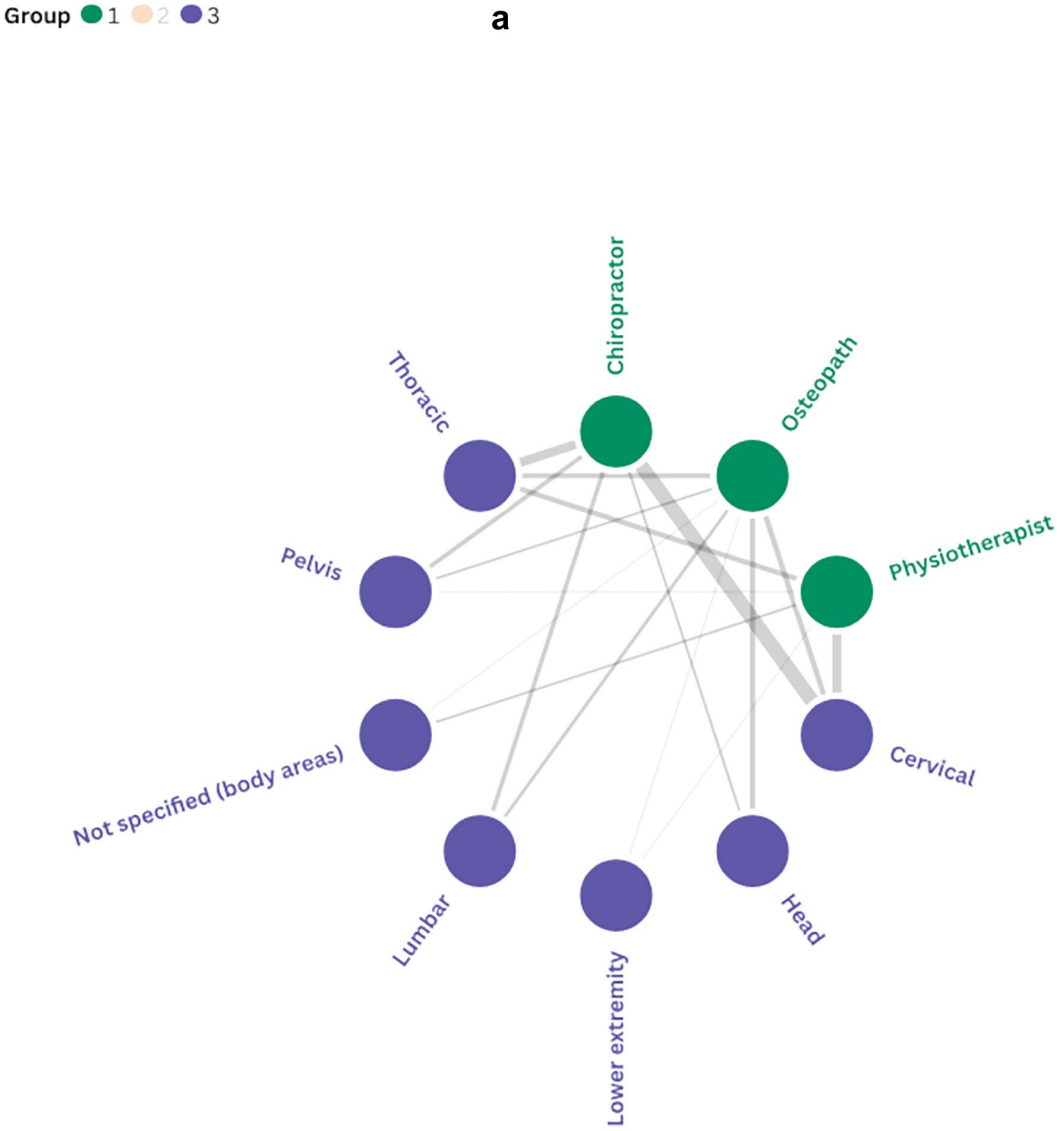

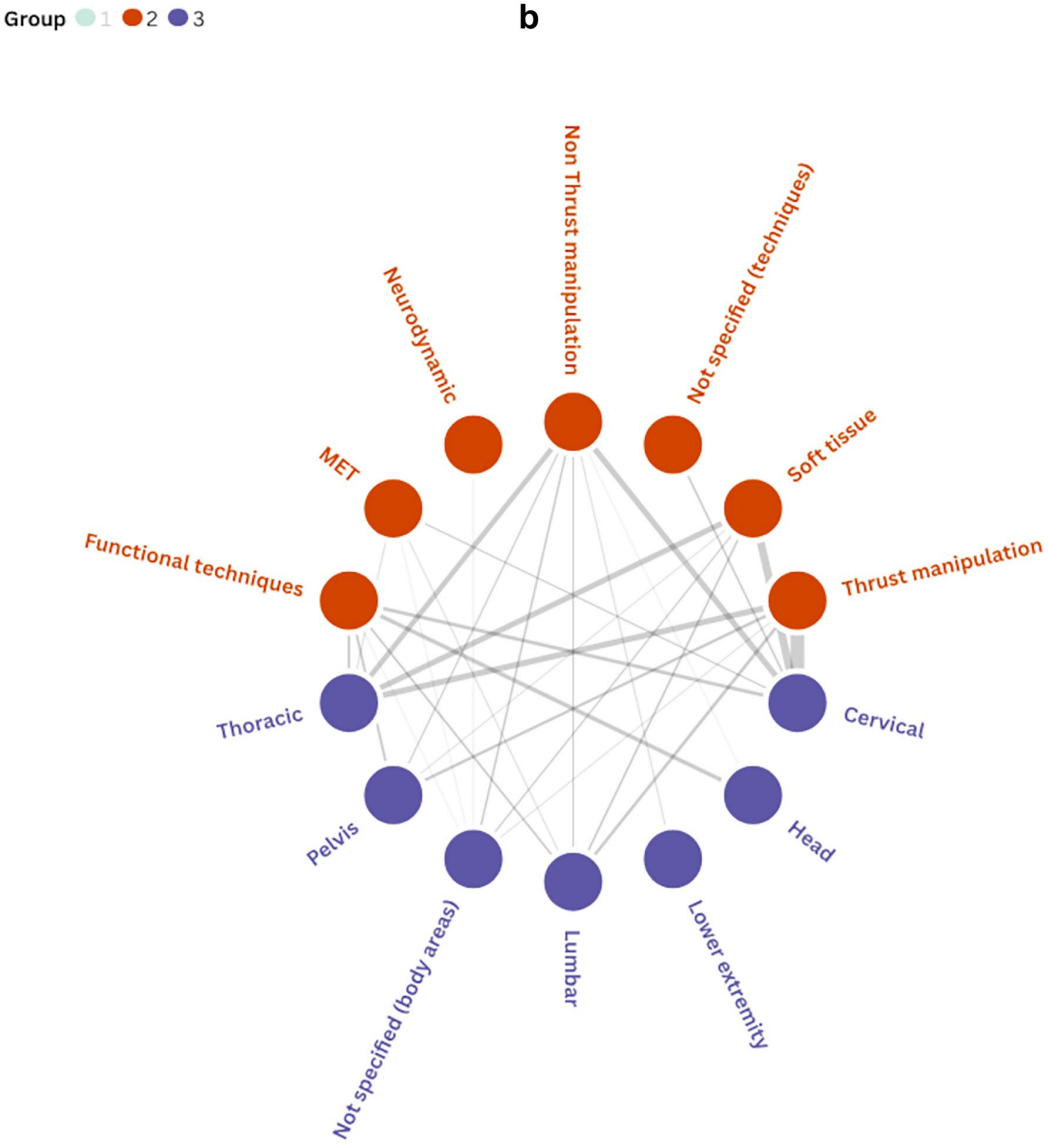

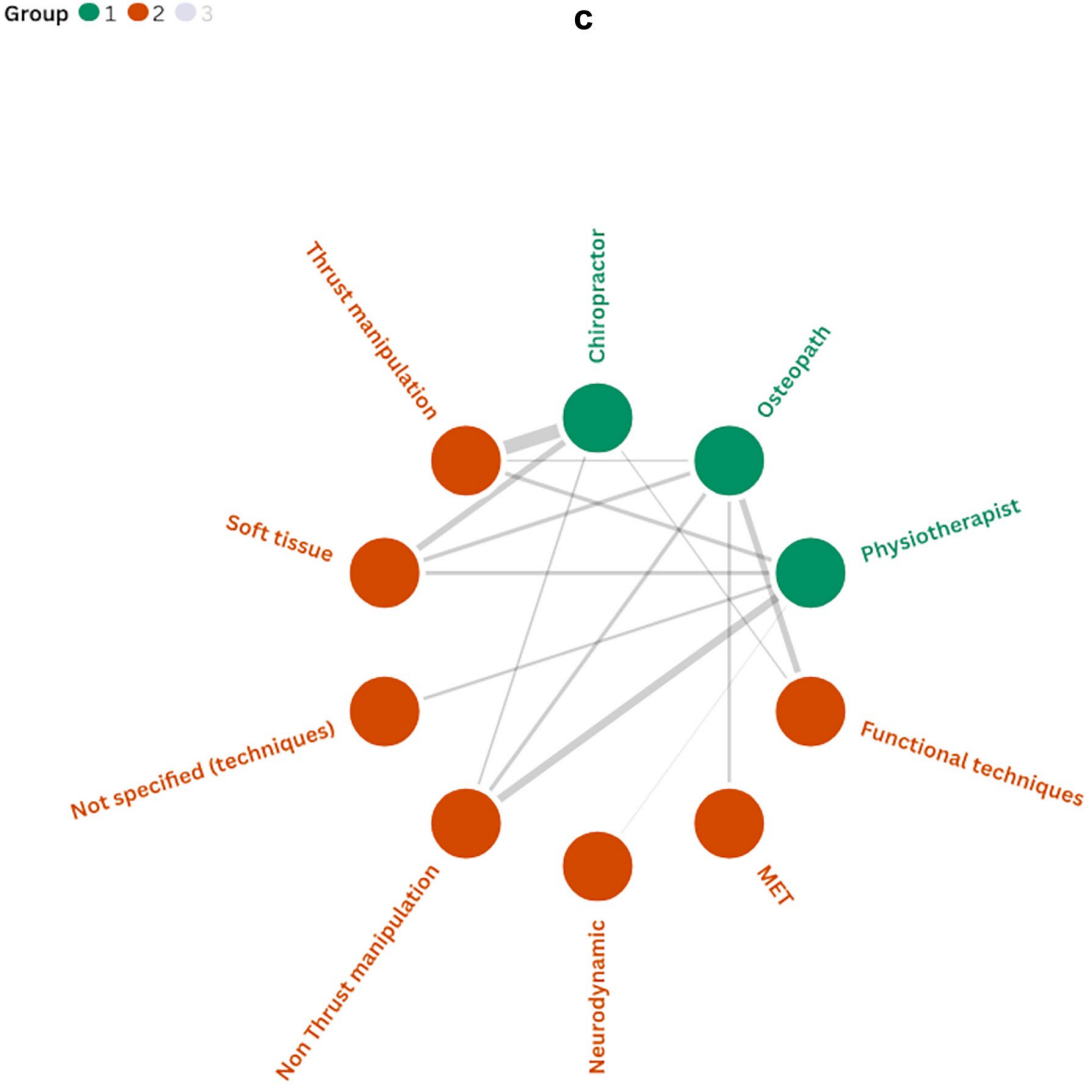
**a** Associations between professional background and body regions treated. **b** Associations between body regions treated and manual therapy techniques. **c** Associations between professional background and manual therapy techniques. Notes: Line thickness indicates the frequency of co-occurrence

As shown in Fig. 5a–c, relationships emerged between professional background, the manual therapy techniques used, and the body regions treated. A positive trend was observed towards the use of thrust and non-thrust manipulation techniques directed at the cervicothoracic region. In contrast, relatively few interventions have focused on the head. Additionally, chiropractors predominantly delivered thrust manipulation while osteopaths and physiotherapists showed more variability in their techniques. Further information on the intervention duration and number of sessions is provided in Additional File 2.

### Manual therapy intervention and combined intervention

**Fig. 6:**
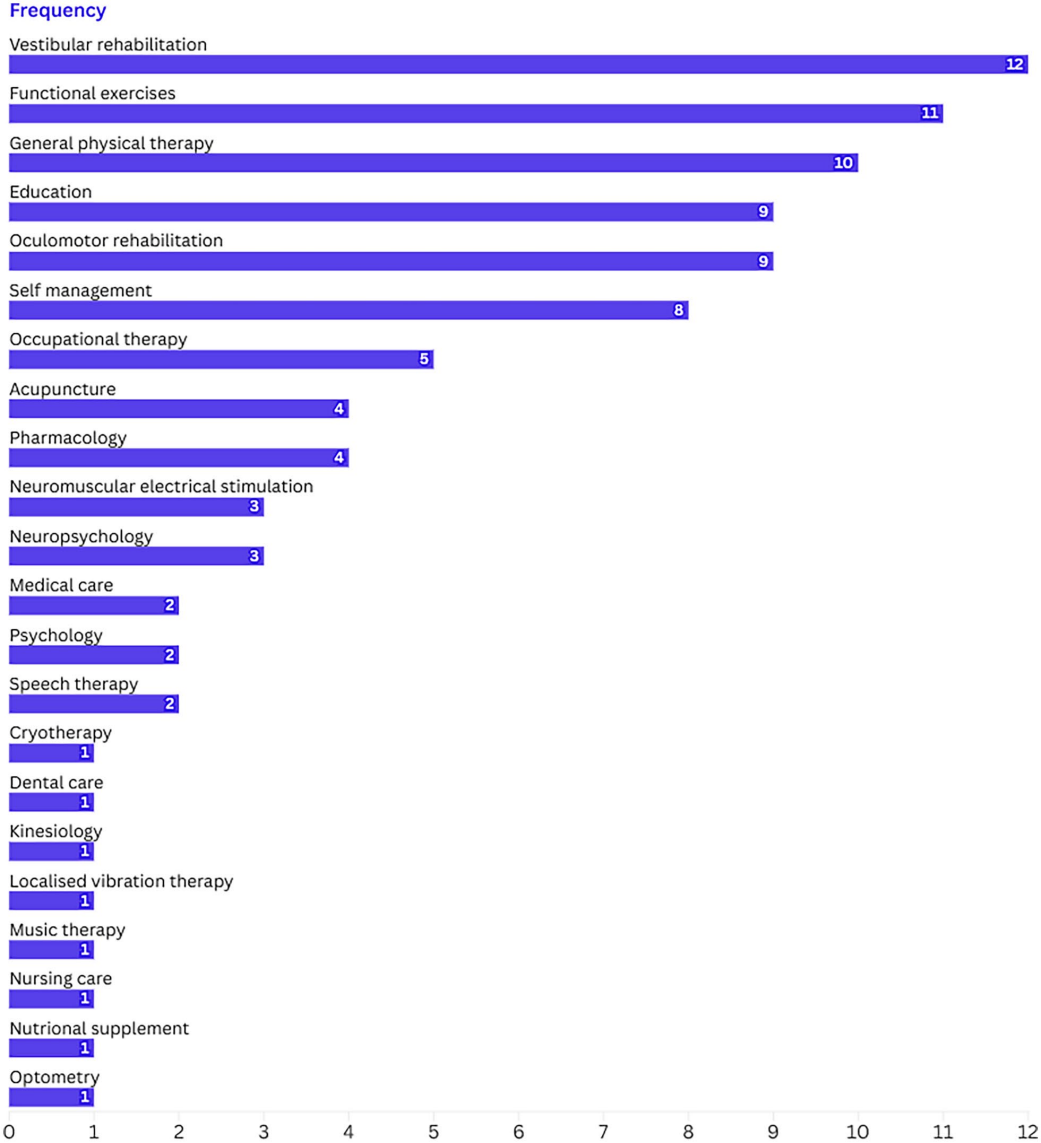
Frequencies of combined interventions with MT in the selected studies

In most eligible studies (n = 27), MT interventions were delivered alongside other therapeutic modalities, including vestibular rehabilitation (n = 12), oculomotor rehabilitation (n = 11), general physiotherapy (n = 10), education (n = 9), functional exercises (n = 9), self-management exercises (n = 8), occupational therapy (n = 5) and psychological support (n = 5). Figure 6 presents the range of therapeutic modalities that were provided alongside MT.

### Adverse events

Adverse events were explicitly reported as an outcome measure in six studies [55, 59, 65, 72, 73, 75]. Across the 42 studies, only 18 clearly addressed the presence or absence of harm. Of these, 16 reported no adverse effects, whereas one study noted mild posttreatment muscle soreness [38]. One study involving participants with moderate to severe TBI in intensive care reported serious adverse events; however, these adverse events were attributed to participants’ underlying health conditions rather than the MT intervention itself [63]. The remaining 24 studies did not report adverse events at all.

### Methodological characteristics of included studies

The included studies displayed a wide range of methodological characteristics. Case reports commonly lacked lack detailed timelines, clearly defined post-intervention outcomes, and reporting of adverse events. Case series presented a heterogenous methodological profile. Some studies provided reliable diagnostic information and clearly defined outcomes, while others demonstrated limitations in statistical analysis and the reporting of participant demographics. RCTs and experimental studies tended to provide more comprehensive reporting overall, although blinding and allocation procedures were often not described or not performed. Among cohort studies, the most common limitations were related to selection procedures and incomplete follow-up reporting. Full results of the appraisal for each study are provided in Additional File 3.

## Discussion

### Brief overview of the results

This scoping review identified and synthesised literature on MT in the management of TBI, with most studies focusing on mild cases in athletic populations. MT interventions—mainly thrust, non-thrust, and soft tissue techniques targeting the cervicothoracic region—are often delivered as part of multimodal rehabilitation to address symptoms such as headache, neck pain, and dizziness. The outcome measures were heterogeneous, and while adverse events were poorly reported, MT appeared safe when documented.

### TBI profile, population and outcome measures

Our findings reflect current research trends, where mild TBI, especially sport-related cases, is studied significantly more frequently than moderate to severe TBI. Given the high prevalence of persistent pain following moderate to severe TBI [77] and the potential of MT as a nonpharmacological alternative to opioid use [78], further research is needed to clarify the role of MT in this population. The included studies focused on interventions provided during the subacute and long-term phases of TBI. A recent RCT investigating the timing of physiotherapy in sport-related mild TBI patients revealed that early intervention during the subacute phase led to improved sensorimotor integration and reduced dizziness compared with delayed care [79]. Although MT was not explicitly examined in that trial, future studies should explore its potential effectiveness during the subacute phase across the full spectrum of TBI severity.

The participants in the reviewed studies showed a clear predominance of adults and individuals involved in sports. This trend reflects broader disparities in the TBI literature, where children, older adults and nonsporting populations remain underrepresented [1]. This is of concern because children and older adults are particularly vulnerable, facing an increased risk of long-term complications following TBI, including cognitive, physical, and functional impairments [80, 81]. Given these risks, further research should examine whether MT may benefit these populations.

The outcome measures used across studies were highly heterogeneous, likely reflecting differences in study design and the diverse symptom profiles seen in individuals with TBI [1]. However, the frequent use of validated instruments such as the RPQ and the SCAT is encouraging, as both are recommended for clinical and research applications [10, 82].

Headaches, dizziness, and neck pain were the most frequently reported and assessed symptoms among participants, which is consistent with the typical physical symptom profile observed following TBI [83, 84]. Although this review did not assess treatment effectiveness, the use of MT appears to extend beyond physical symptoms, including those in the cognitive, emotional and sleep-related domains. This broader scope of MT application may be partly explained by underlying physiological mechanisms. A recent systematic review reported preliminary evidence that MT may positively influence mental health outcomes by modulating autonomic nervous system (ANS) activity, as measured by HRV and skin conductance [85]. This mechanism is further supported by a study demonstrating changes in ANS function in mild TBI patients following cervical MT [56]. ANS dysregulation is a recognised feature of TBI across all severity levels [86, 87] and may contribute to persistent symptoms. This plausible pathway through which MT could impact not only physical symptoms, but also cognitive, emotional and sleep-related domains, requires further research.

### Manual therapy, combined interventions and adverse events

MT interventions primarily include thrust and non-thrust manipulation, as well as soft tissue techniques targeting the cervical and thoracic spine. This focus is supported by international guidelines [10] and current evidence showing that cervical dysfunction can contribute to persistent symptoms, including headache, dizziness and neck pain [88]. In line with systematic reviews suggesting a multidisciplinary approach in mild TBI care [89], our findings revealed that MT was frequently integrated into other treatment modalities, including vestibular and oculomotor rehabilitation, exercise and education. This collaborative approach reflects the growing recognition that complex symptom presentations are best addressed through interdisciplinary care, which is an aspect that is increasingly valued by patients with persistent symptoms [90].

Headache was the most frequently reported symptom among participants in the included studies, which is consistent with its high prevalence following TBI [91]. A recent review of the pathophysiology of posttraumatic headache highlighted the potential role of both peripheral and central sensitisation mechanisms [92]. MT is hypothesised to produce analgesic effects through a combination of contextual factors and complex multisystem processes, including the modulation of neuroimmune, neuroendocrine and autonomic pathways [16, 93]. Despite the high frequency of headache, relatively few MT interventions have specifically targeted the head. Well-designed, proof-of-concept trials are warranted to explore the safety, feasibility, and potential therapeutic value of MT applied directly to the head in individuals experiencing posttraumatic headache.

Adverse events were not reported in most of the included studies (24/42). This is concerning given that documentation on patient safety is a fundamental requirement of clinical research [94]. The potential complications associated with TBI in moderate to severe cases warrant the cautious application of MT in the acute phase [14]. Among the studies reporting harm (18/42), only 2 studies noted any events: one noted minor muscle soreness, which is consistent with the literature [95], whereas the other involved serious adverse events likely attributable to the patients’ critical health status rather than MT intervention. Future studies should implement systematic monitoring and clear reporting of safety outcomes.

### Methodological characteristics and reporting

The included studies presented several reporting and methodological limitations. The case reports and case series lacked adherence to established reporting guidelines, particularly in areas such as patient’s history timeline and reporting of adverse events [96]. While RCTs generally offered more structured reporting, challenges related to blinding procedures were commonly observed. Recent guidance on the design and reporting of control interventions in nonpharmacological research may support improvements in future trials [97].

## Limitations

This scoping review has several limitations. Studies published before 2010 were excluded; however, given the number and relevance of included sources, we believe that this did not significantly affect the results. Only studies published in English or French were considered, which may have limited the pool of eligible studies, although no articles were excluded solely because of language. To maintain a specific focus on TBI, studies on acquired brain injury (ABI) were excluded. While the ABI encompasses conditions such as stroke and severe TBI, this decision may have resulted in the omission of relevant data concerning overlapping populations. Heterogeneity in participants’ characteristics, symptom profiles, outcome measures, and MT interventions poses challenges for direct comparison. One perspective for future research could be to examine the effects of MT according to different concussion subtypes, as recently proposed in work on sport-related concussion [98, 99]. Identifying whether certain subtypes are more responsive to MT would help refine clinical application and guide more targeted research. However, we believe that this review provides a clear and representative overview of the current landscape of MT in TBI management.

## Conclusions

This scoping review mapped the existing literature on the use of MT in the management of TBI. Most studies focused on mild TBI, particularly in sport-related contexts, and commonly investigated MT interventions such as thrust, non-thrust and soft tissue techniques, primarily targeting physical symptoms like headache, neck pain and dizziness. A smaller proportion of studies examined MT in relation to cognitive, emotional, or sleep-related symptoms, highlighting an area in need of further exploration. Future research should investigate potential mechanisms of MT, examine how it might be integrated into interdisciplinary care across different TBI severities and populations, and improve methodological reporting, particularly regarding adverse events.

## Supplementary Information

Supplementary Material 1

Supplementary Material 2

Supplementary Material 3

## Data Availability

Data is provided within the manuscript and supplementary information files.

## Abbreviations

ANS: Autonomic nervous system
COPs: Chiropractors, osteopaths, physiotherapists
HRV: Heart rate variability
ImPACT: Immediate post-concussion assessment and cognitive testing
JBI: Joanna Briggs Institute
MT: Manual therapy
MSK: Musculoskeletal
PCC: Population, concept, context
PCSS: Post Concussion Symptom Scale
PRISMA-ScR: Preferred Systematic Reviews and Meta-Analysis Protocols, Scoping Reviews extension
RCTs: Randomised controlled trials
SCAT: Sport concussion assessment tool
TBI: Traumatic brain injury
RPQ: Rivermead Post-Concussion Questionnaire
