# Supplementary material for: Exploring the use of manual therapy in the management of traumatic brain injury: a scoping review
Source: Chiropr Man Therap. 2025 Oct 10;33:42. doi: 10.1186/s12998-025-00606-y (PMC12512715; doi:10.1186/s12998-025-00606-y)
Supplement: Supplementary file 1 — Supplementary Material 1 [file 12998_2025_606_MOESM1_ESM.docx]

PubMed database search strategy:

Search date: 02/07/2024

(("Physical Therapy Modalities"[MeSH Terms] OR "manual therap*"[Text Word] OR "manipulative therap*"[Text Word] OR "physiotherap*"[Text Word] OR "physiotherapy technique*"[Text Word] OR "physical therap*"[Text Word] OR "chiropractic manipulation*"[Text Word] OR "chiropractic adjustment*"[Text Word] OR "osteopathic manipulative treatment*"[Text Word] OR "osteopathic manipulation*"[Text Word] OR "Chiropractic"[Text Word] OR "Chiropractic Management"[Text Word] OR "Osteopathic"[Text Word] OR "Osteopathic Management"[Text Word]) AND ("humans"[MeSH Terms] AND 2010/01/01:2024/07/02[Date - Publication] AND ("english"[Language] OR "french"[Language])) AND (("Brain Injuries"[MeSH Terms] OR "head injuries, closed"[MeSH Terms] OR "traumatic brain injur*"[Text Word] OR "TBI"[Text Word] OR "severe traumatic brain injur*"[Text Word] OR "moderate traumatic brain injur*"[Text Word] OR "mild traumatic brain injur*"[Text Word] OR "Concussion"[Text Word] OR "post concussion syndrome*"[Text Word] OR "post concussion symptom*"[Text Word] OR "persistent post concussion syndrome*"[Text Word] OR "persistent post concussion symptom*"[Text Word] OR "chronic post concussive syndrome*"[Text Word] OR "chronic post concussive symptom*"[Text Word] OR "head injur*"[Text Word]) AND ("humans"[MeSH Terms] AND 2010/01/01:2024[Date - Publication] AND ("english"[Language] OR "french"[Language])))) AND ((humans[Filter]) AND (2010:2024/7/2[pdat]) AND (english[Filter] OR french[Filter]))
