# Supplementary material for: Exploring the use of manual therapy in the management of traumatic brain injury: a scoping review
Source: Chiropr Man Therap. 2025 Oct 10;33:42. doi: 10.1186/s12998-025-00606-y (PMC12512715; doi:10.1186/s12998-025-00606-y)
Supplement: Supplementary file 3 — Supplementary Material 3 [file 12998_2025_606_MOESM3_ESM.docx]

|  | 1. Patient’s demographic characteristics clearly described | 2. Patient’s history presented as a timeline | 3. Current clinical condition clearly described | 4. Diagnostic tests and results clearly described | 5. Intervention(s) clearly described | 6. Post-intervention clinical condition clearly described | 7. Adverse events reported | 8. Takeaway lessons |
| --- | --- | --- | --- | --- | --- | --- | --- | --- |
| Angerami and Evans, 2023 | Yes | No | Unclear | Yes | Unclear | Unclear | No | No |
| Bloink and Blum, 2021 | Unclear | No | Yes | Unclear | Unclear | Unclear | No | Unclear |
| Castillo et al., 2016 | No | No | Yes | Yes | Unclear | Yes | Yes | Yes |
| Chung, 2019 | No | No | No | Unclear | Yes | Unclear | Yes | Unclear |
| Eifertet al., 2013 | No | No | Yes | Yes | Yes | Yes | Yes | Yes |
| Gergen, 2015 | Unclear | Yes | Yes | Unclear | Yes | Unclear | No | Yes |
| Guernsey et al., 2016 | No | No | Unclear | Unclear | Yes | Yes | No | Yes |
| Holbeck and Blum, 2021 | Yes | Unclear | Yes | No | Unclear | Unclear | No | Unclear |
| Hunt et al., 2018 | No | No | Yes | Unclear | Yes | Unclear | No | Yes |
| Marshall et al., 2015 | No | No | Yes | Unclear | Yes | Yes | No | Yes |
| McArthur and Olson, 2019 | Unclear | Unclear | Yes | Yes | Yes | Yes | No | Yes |
| McCallister et al., 2016 | Yes | No | Yes | Yes | Yes | Yes | Yes | Yes |
| Null and Null, 2019 | Unclear | No | Unclear | Unclear | Yes | Unclear | No | Unclear |
| Olson et al., 2016 | Unclear | Unclear | Yes | Yes | Unclear | Unclear | No | Yes |
| Olson et al., 2018 | Unclear | No | Yes | Yes | Yes | Yes | No | Yes |
| Olson et al., 2020 | No | No | Yes | Unclear | Yes | Unclear | No | Unclear |
| Patel, 2020 | Unclear | Unclear | Yes | Yes | Yes | Yes | No | Yes |
| Remata and Blum, 2021 | No | No | No | No | No | Unclear | No | Unclear |
| Wasylynko, 2017 | Yes | Unclear | Yes | No | Yes | No | No | Yes |

*Assessment of methodological characteristics and reporting – Case reports*

*Note: Colour coding reflects the assessment of methodological criteria and reporting. Green indicates the criterion was clearly met and reported; yellow indicates it was partially met or unclear; red indicates it was not met or not reported*

|  | 1. Clear inclusion criteria | 2. Condition reliably measured for all participants | 3. Valid methods used for identification of the condition for all participants | 4. Consecutive inclusion of participants | 5. Complete inclusion of participants | 6.Reporting participants demographics | 7. Reporting clinical information of the participants | 8. Outcomes of cases clearly reported. | 9.Reporting of presenting site(s)/clinic(s) demographic information | 10. Appropriate statistical analysis |  |
| --- | --- | --- | --- | --- | --- | --- | --- | --- | --- | --- | --- |
| Chappell et al., 2015* | Yes | Yes | Yes | No | Unclear | No | No | Unclear | Yes | Yes | *Retrospective chart review |
| Corten et al., 2020 | Yes | Yes | Yes | Yes | No | Yes | Yes | Yes | Yes | Yes | *Retrospective folder review |
| Germann et al., 2020 | No | Yes | Yes | No | Unclear | No | Yes | Yes | No | No |  |
| Hammerle et al., 2023 | No | Yes | Yes | No | Unclear | Yes | Yes | Yes | Yes | No |  |
| Kennedy et al., 2017 | Yes | Yes | Yes | Yes | Yes | Yes | Yes | Yes | Yes | Yes |  |
| Kennedy et al., 2021 | Yes | Yes | Yes | Yes | Unclear | Yes | Yes | Yes | Yes | Yes |  |
| Moore, 2019 | Unclear | Yes | Yes | Yes | No | No | Unclear | Yes | No | No |  |
| Nguyen et al., 2023* | Yes | Yes | Yes | Yes | Unclear | Yes | Yes | Yes | Yes | Yes | *Single-case experimental design |
| Patel and Sabini, 2018* | Yes | Yes | Yes | Yes | No | Yes | Yes | Yes | No | No | *Prospective observational pilot study |

*Assessment of methodological characteristics and reporting – Case series*

*Note: Colour coding reflects the assessment of methodological criteria and reporting. Green indicates the criterion was clearly met and reported; yellow indicates it was partially met or unclear; red indicates it was not met or not reported*

|  | 1. Randomisation | 2. Treatment allocation | 3. Baseline similarity | 4. Participant blinding | 5. Provider blinding | 6. Groups treated identically other than intervention of interest | 7. Assessor blinding | 8. Outcomes measured in the same way | 9. Outcome measurement reliability | 10. Follow up | 11. Intention to Treat Analysis | 12. Appropriate statistical analysis | 13. Appropriate trial design |
| --- | --- | --- | --- | --- | --- | --- | --- | --- | --- | --- | --- | --- | --- |
| Cade et al., 2024 | Yes | Yes | Yes | Yes | No | Yes | Yes | Yes | Yes | Yes | Yes | Yes | Yes |
| Esterov et al., 2021 | No | No | Unclear | No | No | Yes | No | No | Unclear | Unclear | Yes | Yes | Yes |
| Farrell et al., 2024 | Yes | Yes | Yes | No | No | Yes | Yes | Yes | Yes | Yes | Yes | Yes | Yes |
| Langevin et al., 2022 | Yes | Yes | Yes | Unclear | No | Yes | Yes | Yes | Yes | Unclear | Yes | Yes | Yes |
| Mancini et al., 2023 | Yes | No | No | No | No | Yes | Yes | Yes | Yes | No | No | Yes | Unclear |
| Reneker et al., 2017 | Yes | Unclear | Yes | Yes | No | Yes | Unclear | Yes | Yes | Unclear | Yes | Yes | Yes |
| Schneider et al., 2014 | Yes | Yes | Yes | Unclear | No | Yes | Yes | Yes | Unclear | Yes | Yes | Yes | Yes |
| Tomar et al., 2019 | Yes | Yes | Yes | Yes | No | Yes | Yes | Yes | Yes | Yes | Yes | Yes | Yes |
| Yao et al., 2020 | Yes | No | Unclear | No | No | Unclear | Unclear | Yes | Unclear | No | No | Yes | Yes |
| Yen et al., 2024 | Yes | Yes | Yes | No | No | Unclear | Yes | Yes | Yes | Yes | Yes | Yes | Yes |

*Assessment of methodological characteristics and reporting – Randomised Controlled Trials*

*Note: Colour coding reflects the assessment of methodological criteria and reporting. Green indicates the criterion was clearly met and reported; yellow indicates it was partially met or unclear; red indicates it was not met or not reported*

|  | 1.Clear cause and effect | 2. Control group | 3. Control participants similarity | 4. Similar treatment/care (apart from intervention) | 5. Multiple measurements | 6. Participants outcomes measured in the same way | 7. Outcomes measured reliably | 8. Follow up complete? | 9. Appropriate statistical analysis |
| --- | --- | --- | --- | --- | --- | --- | --- | --- | --- |
| Cramer et al., 2010 | Yes | No | Yes | Yes | Yes | Yes | Yes | Unclear | No |
| Langevin et al., 2024 | Yes | Yes | No | Yes | Yes | Yes | Yes | No | Yes |

*Assessment of methodological characteristics and reporting – Experimental studies*

*Note: Colour coding reflects the assessment of methodological criteria and reporting. Green indicates the criterion was clearly met and reported; yellow indicates it was partially met or unclear; red indicates it was not met or not reported*

|  | 1.Similar groups | 2. Exposures measured similarly between groups | 3. Exposure measured reliably | 4. Confounding factors | 5. Strategies to deal with confounding factors | 6.Participants free of the outcome at the start of the study | 7.Outcomes measured reliably | 8. Follow-up time reported and long enough | 9. Follow up | 10. Strategies to address incomplete follow up | 11. Appropriate statistical analysis |
| --- | --- | --- | --- | --- | --- | --- | --- | --- | --- | --- | --- |
| Grabowski et al., 2017 | ***** | ***** | **Unclear** | **Unclear** | **Unclear** | **Yes** | **Unclear** | **Unclear** | **Yes** | **No** | **Unclear** |
| Wong et al., 2021 | ***** | ***** | **Unclear** | **No** | **No** | **No** | **Yes** | **Unclear** | **No** | **No** | **Unclear** |

*Assessment of methodological characteristics and reporting – Cohort studies
*Not applicable due to the retrospective nature of this cohort study*

*Note: Colour coding reflects the assessment of methodological criteria and reporting. Green indicates the criterion was clearly met and reported; yellow indicates it was partially met or unclear; red indicates it was not met or not reported*
