# Supplementary material for: Exploring the use of manual therapy in the management of traumatic brain injury: a scoping review
Source: Chiropr Man Therap. 2025 Oct 10;33:42. doi: 10.1186/s12998-025-00606-y (PMC12512715; doi:10.1186/s12998-025-00606-y)
Supplement: Supplementary file 4 [file 12998_2025_606_MOESM2_ESM.odt]

| Authors, year and country | Study design, sample size and patient characteristic | TBI severity and injury phase | TBI symptoms addressed | Profession, Setting, Number of sessions | Manual therapy intervention and body area treated | Combined intervention | Comparator | Outcome measures used | Side effects measured and reported? |
| --- | --- | --- | --- | --- | --- | --- | --- | --- | --- |
| Angerami and Evans, 2023 USA |  | To evaluate and discuss the effects of chiropractic intervention on a patient with post concussive symptoms following a sport induced concussion. |
| Case report, N = 1, 20-year-old male, athlete | Mild, Subacute | Headache, nausea, dizziness, photophobia, vision difficulties, memory difficulties, concentration difficulties, sleeping difficulties and fatigue. | Chiro, Private clinic, 9 sessions over 5 weeks | Thrust manipulation; Cervical, thoracic, lumbar, pelvis | Self-management; neuromuscular electrical stimulation | NA | Visual Analogue Scale, Self-reported improvement | No |
| Bloink and Blum, 2021 USA |  | To evaluate and discuss the effects of chiropractic intervention on a patient with post concussive symptoms following a sport induced concussion. |
| Case Report, N = 1, 21 years old female, Adult non athlete | Mild, Long term | Dizziness, mental confusion, nausea, headache, photophobia, and vision difficulties | Chiro, Private clinic, 9 sessions | Thrust manipulation, non-thrust manipulation;  Cervical, TMJ | Dental Care | NA | Self-reported improvement | No |
| Cade and Turnbull, 2024 New Zealand |  | To investigate whether a chiropractic intervention intended at reducing spinal proprioceptive dysregulation can alter some of the commonly reported defects in eye-tracking function and spatial awareness that occur following mild TBI |
| RCT, Chiro = 20 Control = 20, Age: 18-35 years (Mean age: 25.8 ± 4.1 in the intervention group; 25.7 ± 4.7 in the control group), Gender: Chiropractic group (15 females), Control group (13 females), Mixed (adult non athletes and adult athletes) | Mild, Long term | Headache, dizziness, nausea, phonophobia, sleeping difficulties, fatigue, irritable, depressed, frustrated, memory difficulties, concentration difficulties, vision difficulties, photophobia, restlessness | Chiro, Educational institutions, 1 session | Thrust manipulation;  Cervical, thoracic, lumbar, pelvis | Standalone MT | Sham MT to cervical, thoracic, lumbar and pelvis | Computerized Eye Tracker Assessments (CEAs) | Yes, but none reported |
| Castillo et al., 2016 USA |  | To illustrate the potential role of osteopathic manipulative treatment in the recovery of prolonged post-concussive symptoms in an adolescent |
| Case Report, N = 1, 16 years old, female, Teenager non athlete | Mild, Long Term | Headache, neck pain, vertigo, dizziness, concentration difficulties, low back pain. | Osteo, Hospitals, 6 weekly sessions | Soft tissue, non-thrust manipulation, functional techniques, MET;  Cervical, lumbar, pelvis, head | Vestibular rehabilitation; self-management; general physical therapy | NA | Post concussion symptom scale (PCSS), Balanced Error Scoring System (BESS), Self-reported improvement | Yes, but none reported |
| Chappell et al., 2015 USA |  | To retrospectively examine the effectiveness of OMT at reducing concussive symptoms in athletes who were diagnosed with a concussion |
| Retrospective chart review, N = 26, mean age 19,56 gender male 64%, female 36%, Mixed (adult athletes and teenager athletes) | Mild, Acute and Subacute | Headache, dizziness, phonophobia, mental confusion, concentration difficulties, fatigue, irritable, depressed | Osteo, Educational institutions, 1 session | Thrust manipulation, non-thrust manipulation, functional techniques, MET, soft tissue;  Not specified | Standalone MT | NA | SCAT2, Self-reported improvement | No |
| Chung, 2019 USA |  | To assess the effect of low- force upper cervical spine chiropractic techniques on post-concussive symptoms in a 16 and a 30-year-old female. |
| Case Report, N = 2, 16-year-old female, 30-year-old female, Mixed (teenager non athlete, adult non athlete) | Mild, Long term | Headaches, dizziness, concentration difficulties, vertigo, neck pain, mental confusion. | Chiro, Private clinic, Patient 1 = 14 sessions with an adjustment on 6 of these. Patient 2 = 17 sessions with adjustments on 10. | Thrust manipulation;  Cervical | Standalone MT | NA | Visual Analogue Scale | Yes, mild soreness reported by patient 1 |
| Corten et al., 2020 South Africa |  | To describe the characteristics, course, and outcomes of children hospitalized with TBI, with a focus on physiotherapy's role in their care. |
| Retrospective folder review, Median age: 5.37 years (IQR: 1.88–7.88 years). Gender: 64.6% male, Children, | Mild, Moderate and Severe, Subacute | Neurological deficit | Physio,  Hospitals, 3 sessions (2.0 - 6.0) | Non thrust manipulation, neurodynamic, soft tissue;  Thoracic, not specified | speech therapy; occupational therapy; self-management; general physical therapy; functional exercises | NA | Adverse events | Yes, but none reported |
| Cramer et al., 2010 USA |  | To determine the safety and effect of pedal pump and thoracic pump techniques on ICP and CPP in patients with severe traumatic brain injuries​. |
| Experimental study, Group 1 (ICP ≤ 20 mm Hg): 13 patients. Group 2 (ICP > 20 mm Hg): 11 patients. Total = 24, Ages 18–69; 15 men, 9 women​. Mean Age: 43.5 years Male Percentage: 62.5% Female Percentage: 37.5% Mixed (adult non athletes and older adults) | Severe,  Unclear | Intracranial pressure, cerebral perfusion pressure | Osteo, Hospitals, Up to 3 sessions | Non thrust manipulation;  Thoracic, Lower extremity | Standalone MT | No comparator group | Intracranial pressure (ICP), Cerebral perfusion pressure (CPP), Adverse events | Yes, but none reported |
| Eifertet al., 2013 Germany |  | To describe and evaluate a program integrating neurorehabilitation into intensive care treatment for patients with severe traumatic brain injuries and disorders of consciousness. The authors used a case report to illustrate this program. |
| Case Report, N = 1, 18 year old male, Adult non athlete | Severe,  Long term | Neurological deficit | Physio, Hospitals 1-2hrs/day for 3 weeks | Non thrust manipulation;  Not specified | General physiotherapy; music therapy; occupational therapy; neuropsychology; pharmacology; nursing therapy | NA | Functional Independence Measure , Disability Rating Scale | Yes, but none reported (the authors present medical complication occurring during the treatment, however, there do not seem related to MT intervention) |
| Esterov, et al., 2021 USA |  | To evaluate the effectiveness and safety of OMT for headaches in patients with PCS. |
| RCT, OMT = 10 Control = 10, Age range: 18–71 years (mean age: 45 in treatment group, 42 in control group). Gender: Treatment group – 7 females, 3 males; Control group – 6 females, 4 males.  Mean Age: 43.5 years Gender Distribution: Females: 65.0%, Males: 35.0%, Mixed (adult non athletes and older adults) | Mild,  Long term | Headache | Osteo, Specialist clinics, 1 session | MET, soft tissue, functional techniques;  Cervical, thoracic | General physiotherapy; occupational therapy; speech therapy; education; pharmacology | Conventional care for concussion management (general physical therapy, occupational therapy, speech therapy, education, pharmacology) | Visual Analogue Scale (headache), Head Impact Test (HIT-6), Adverse events | Yes, but none reported |
| Farrell et al., 2024 New Zealand |  | To evaluate the differential effects of upper and lower cervical spine mobilization on the stress response in individuals with PPCS |
| RCT, Upper cervical mobilization: n=10. Lower cervical mobilization: n=9 Males, aged 19–35 years (mean age: 23.60 for upper group, 25.56 for lower group). Adult non athletes | Mild, Long term | Neck pain, headache, dizziness, nausea, phonophobia, sleeping difficulties, fatigue, irritable, depressed, frustrated, memory difficulties, concentration difficulties, vision difficulties, photophobia, restlessness | Physio, Educational institutions, 1 session | Thrust manipulation;  Cervical | Standalone MT | NA in Proof of Concept Trial. To control for potential contextual influences, each intervention was performed in the same room, between 1pm and 5pm. Temperature was accounted for, and no natural light was allowed to enter the room. | Salivary cortisol concentration (sCOR), Heart rate variability (HRV) using the rMSSD metric, RPQ | Yes, but none reported |
| Gergen, 2015 USA |  | To describe chiropractic and acupuncture care of a patient with acute mild traumatic brain injury (mTBI) symptoms. |
| Case Report, N = 1, 31-year-old female, Adult non athlete | Mild Subacute | Neck pain, headache, nausea, dizziness, tinnitus, fatigue, mental confusion | Chiro, Private clinic, 8 visits over 2.5 weeks | Thrust manipulation, soft tissue;  Cervical | Cryotherapy; acupuncture | NA | Numeric Pain Scale (Neck pain, Headache), Self-reported improvement, Return to play | No |
| Germann et al., 2020 Canada |  | To describe the efficacy of individualized, multi-modal treatment plans delivered by chiropractic sports specialists for managing acute, sub-acute, and chronic post-concussion symptoms (PCS). |
| Case series, N = 3, female 15 years old female 14 years old female 27 years old, Mixed (teenager athletes, adult non athlete) | Mild, Acute, Subacute and Long term | Neck pain, headache, dizziness, phonophobia, concentration difficulties, fatigue, mental confusion | Chiro, Private clinic, case 1: 16 sessions over 8 weeks, case 2: 8 sessions over 5 weeks, case 3: 4 sessions over 4 weeks | Soft tissue, thrust manipulation; Cervical, thoracic | Education; neuromuscular electrical stimulation; acupuncture; vestibular rehabilitation | NA | Neck Disability Index, Post Concussion Symptom Scale (PCSS) | No |
| Grabowski et al., 2017 USA |  | To assess the implementation, safety, and feasibility of a multimodal physical therapy approach in PCS patients. |
| Retrospective cohort study, N = 25, Age: 12–20 years. mean age: 15 Gender: Mixed (11 male, 14 female), Mixed (Teenager athletes and adult athletes) | Mild,  Subacute and Long term | Headache, concentration difficulties, memory difficulties, dizziness, phonophobia, photophobia, restlessness | Physio, Hospitals, Median number of sessions is 4 with a range from 2 to 16 sessions | Thrust manipulation, soft tissue, non-thrust manipulation;  Cervical, thoracic | General physiotherapy; vestibular rehabilitation; oculomotor rehabilitation; functional exercises | NA | Post Concussion Symptom Scale (PCSS), Balance Error Scoring System (BESS), Adverse events | Yes, but none reported |
| Guernsey et al., 2016 USA |  | To decribe the effect of osteopathic manipulative treatment on concussion symptoms and balance in a single patient. |
| Case Report, N = 1, 27 years old, male, Adult athlete | Mild, Subacute | Dizziness, nausea, mental confusion, tinnitus, headache | Osteo, Educational institutions, 1 session | Functional techniques, non-thrust manipulation, soft tissue;  Head, cervical, thoracic, lumbar, pelvis | Standalone MT | NA | Sensory Organization Test (SOT), Self-reported improvement | No |
| Hammerle et al., 2023 USA |  | To describe a biomechanical intervention targeting the upper cervical spine in alleviating symptoms of persistent post-traumatic headache and intermittent glossopharyngeal nerve irritation. |
| Case series, N = 3, 48-year-old male 25-year-old female 45-year-old female mean age = 39.33, Mixed (2 militaries and 1 adult non athlete) | Mild, Long term | Headache, dizziness, autonomic dysfunction, dysphagia, throat irritation, upper pharyngeal collapse, vision difficulties, eye dryness | Physio, Hospitals, Patient A: 26 sessions over 72 weeks Patient B: 15 sessions over 36 weeks Patient C: 13 sessions over 16 weeks | Non thrust manipulation, thrust manipulation;  Cervical, thoracic, pelvis | Education; self-management; functional exercises | NA | Numeric Pain Scale (Headache), Composite Autonomic Symptom Score-31 (COMPASS) | Yes, but none reported |
| Holbeck and Blum, 2021 USA |  | To examine the effectiveness of chiropractic sacro occipital and cranial techniques for managing post-concussion syndrome symptoms in a teenage athlete |
| Case Report, N = 1, 14 years old female, Teenager athlete | Mild, Long term | Memory difficulties, vision difficulties, dizziness, nausea, fatigue, headache | Chiro, Private clinic, 5 sessions | Functional techniques, thrust manipulation; Head, cervical, pelvis | Standalone MT | NA | Self-reported improvement, Romberg test | No |
| Hunt et al., 2018 New Zealand |  | To describe the effects of a course of chiropractic treatment on a 13-year-old adolescent with mild TBI. |
| Case Report, N = 1, 13-year-old male, Teenager non athlete | Mild, Subacute | Headache, concentration difficulty, neck pain, sensitivity to light and sound | Chiro, Private clinic, 8 sessions over 5 weeks | Thrust manipulation;  Cervical, thoracic, pelvis | Standalone MT | NA | Visual Analogue Scale (headache, concentration), Self-reported improvement | No |
| Kennedy et al., 2017 New Zealand |  | To describe cervical spine findings and outcomes of physiotherapy treatment in patients with persistent post-concussion symptoms and outline clinical characteristics of cervicogenic symptoms. |
| Retrospective case series, 2 groups with (N=32) or without cervicogenic component (N=14), Mean age: 26.5 years (Range: 12–62, SD: 12.0) Male: 21 (46%), Mixed (teenager non athletes, adult non athletes and adult athletes) | Mild,  Subacute and Long term | Headache, dizziness, neck pain | Physio, Private clinic, Mean: 5.2 sessions (Range: 2–12) | Not specified;  Cervical | Acupuncture; functional exercises | NA | Patient Specific Functional Scale (PSFS), Numeric Pain Scale, Active Range of Motion (Neck) | No |
| Kennedy et al., 2021 New Zealand |  | To describe the individual long-term outcomes of people with persistent symptoms following a concussion who received neck treatment within a multidisciplinary concussion service and to report how participants describe the outcomes of neck treatments. |
| Case series, N = 11, Mean age = 36 years (SD = 17); 64% female, 36% male, Mixed (adult non athletes and 1 older adult) | Mild,  Subacute and Long term | Neck pain, headache, dizziness, nausea, phonophobia, sleeping difficulties, fatigue, irritable, depressed, frustrated, memory difficulties, concentration difficulties, vision difficulties, photophobia, restlessness | Physio, Specialist clinics, Median = 4.5 sessions (range: 1–9) | Not specified;  Cervical | Vestibular rehabilitation; self-management; occupational therapy; oculomotor rehabilitation; education; neuropsychology; functional exercises | NA | RPQ, Neck Disability Index, Dizziness Handicap Inventory (DHI), Self-reported improvement, Standardized Physical Examination | Yes, but none reported |
| Langevin et al., 2022 Canada |  | To determine if adding cervicovestibular rehabilitation to SLAE improves symptoms and clinical recovery in adults with mTBI compared to SLAE alone |
| RCT, Experimental Group (cervicovestibular rehabilitation + SLAE) = 30 Control Group (SLAE alone) = 30 Number of Participants: 60 Age: Mean = 39 years (weighted average of both groups) Gender: 68.3% women (41/60) Mixed (Adult non athletes and adult athletes) | Mild,  Subacute and Long term | Headache, dizziness, neck pain, phonophobia, nausea, mental confusion, concentration difficulties, memory difficulties | Physio, Private clinics; Educational institutions, 8 treatment sessions over 6 weeks and 5 evaluation sessions over 26 weeks | thrust manipulation, non-thrust manipulation, soft tissue;  Cervical | Education; neuropsychology; kinesiologist; general physiotherapy; functional exercises; vestibular rehabilitation; oculomotor rehabilitation | Symptom-limited aerobic exercise program which included neuropsychology, kinesiologist and education | Post Concussion Symptom Scale (PCSS), Neck Disability Index, Headache Disability Inventory (HDI), Dizziness Handicap Inventory (DHI), Numeric Pain Scale (Headache, Neck pain), Global Rating of Change (GRC), Active Range of Motion (e.g., Flexion-Rotation Test),  Head Impulse Test (HIT) for vestibular and oculomotor impairments Standardised Physical Examination, Vestibular Ocular Motor Screening (VOMS | Yes, but none reported |
| Langevin et al., 2024 Canada |  | To compare the effects of a cervicovestibular telerehabilitation program (advice, education, no manual techniques) combined with sub-symptom threshold aerobic exercise (STAE) program to a similar in-clinic cervicovestibular telerehabilitation program (advice, education, home exercises, manual techniques) combined with a STAE program in adults with persisting PCS on the severity of symptoms and disability. |
| Experimental study, Intervention (in-clinic) = 30 telerehabilitation = 11, Mean Age: 38.6 years. Gender: 68.5% female, 31.5% male, Mixed (Adult athletes and adult non athletes) | Mild, Subacute and Long term | Headache, dizziness, neck pain, phonophobia, nausea, mental confusion, concentration difficulties, memory difficulties | Physio, Private clinics; Educational institutions, 8 sessions over 6 weeks for both groups | Non thrust manipulation, soft tissue;  Cervical | General physiotherapy; vestibular rehabilitation; oculomotor rehabilitation; functional exercises; self-management; education | Telerehabilitation including: cervicovestibular rehabilitation exercise, education, and sub-symptom threshold aerobic exercise | Post concussion symptom scale (PCSS), Neck disability index, Headache Disability Inventory (HDI), Dizziness Handicap Inventory (DHI), Numeric Pain Scale (Headache, Neck pain) | No |
| Mancini et al., 2023 USA |  | To determine if osteopathic manipulative medicine is more effective than education to help patients with new onset impairment (NOI) of neurological functions identified by Immediate Post Concussion Assessment and Cognitive Testing (ImPACT) |
| RCT, OMM = 11 Concussion education = 9, mean age of 20 (±1.8)  OMM group = 3 women (27.3%) 8 men (72.7%) Concussion education group = 3 women (33.3%) 6 men (66.7%), Adult athletes | Mild, Subacute | Headache, memory difficulties, vision difficulties, nausea, dizziness, sleeping difficulties, photophobia, phonophobia, mental confusion, tinnitus | Osteo, Educational institutions, 3 sessions | Functional techniques, MET, thrust manipulation;  Head, cervical, thoracic, lumbar, pelvis | Standalone MT | Education | ImPACT score, King-Devick (KD) Test (visual spatial test cards) | Yes, but none reported |
| Marshall et al., 2015 Canada |  | To describe the role of cervical spine on post-concussion symptoms and highlight the role of cervical spine treatment through a short case series of 5 patients |
| Case Report, N = 5 mean age: 34,6 (range 19-59), gender: male 80% (n=4), female 20% (n=1), Mixed (adult non athletes and adult athletes) | Mild,  Long term | Headache, dizziness, phonophobia, anxiety, vision difficulties, fatigue, photophobia, mental confusion, concentration difficulties, irritable, memory difficulties, neck pain | Chiro, Private clinic, 4 to 26 sessions | Soft tissue, non-thrust manipulation, thrust manipulation;  Cervical | General physiotherapy; vestibular rehabilitation; oculomotor rehabilitation; optometrist; localized vibration therapy | NA | Self-reported improvement (pain, function), post-concussion symptom scale (PCSS) | No |
| McArthur and Olson, 2019 USA |  | To demonstrate the role of chiropractic care, including spinal manipulation and rehabilitation, in concussion recovery and return-to-play processes |
| Case Report, N = 1, 17-year-old, male, Teenager athlete | Mild, Subacute | Headache, nausea, dizziness, concentration difficulties, mental confusion | Chiro, Private clinic, 6 sessions in total, but only first four sessions included manual therapy | Soft tissue, thrust manipulation;  Cervical, thoracic | Self-management; vestibular rehabilitation; oculomotor rehabilitation; education; neuromuscular electrical stimulation | NA | ImPACT score, SCAT 5, Self-reported improvement | No |
| McCallister et al., 2016 |  | To explore the potential benefits and risks of osteopathic manipulative treatment (OMT) in managing somatic dysfunction associated with acute severe traumatic brain injury (TBI). |
| Case Report, N = 2, Case 1: 21-year-old male Case 2: 54-year-old male, Adult non athletes | Severe, Subacute | Proptotic eye, headache, dizziness | Osteo, Hospitals, Case 1: 24 sessions over 42 days Case 2: 5 sessions over 8 days | Functional techniques, soft tissue; Head, cervical, thoracic | Standalone MT | NA | GCS, Somatic Dysfunction, Self-reported improvement | Yes, but none reported |
| Moore, 2019 Canada |  | To describe the chiropractic management of 6 consecutive patients with post-concussion syndrome and to discuss the importance of correcting and maintaining the alignment of the cranio-cervical junction in post-concussion syndrome |
| Case series, N = 6, Mean Age: 55.83 years (ranging from 39 to 82) Gender: 33.33% male, 66.67% female, Mixed (Adult non athletes, adult athletes, older adults) | Mild, Long term | Headache, photophobia, phonophobia, anxiety, nausea, tinnitus, mental confusion, fatigue, sleeping difficulties, dizziness, neck pain, shoulder pain, concentration difficulties, eye pain, dyspnoea, nausea, vision difficulties | Chiro, Private clinic, Mean Number of Sessions: 11.17 sessions over to 12 weeks | Thrust manipulation;  Cervical | Standalone MT | NA | RPQ, Neck Disability Index, Headache Disability Index, Dizziness Handicap Inventory, X-ray | No |
| Nguyen et al., 2023 Australia |  | This pilot study aimed to explore the feasibility and preliminary efficacy of an interdisciplinary intervention for PPCSs |
| Single case experimental design, N = 15, 53% female - mean age 38.3 years, Mixed population (adult athletes, older adults, adult non athletes) | Mild, Long term | Headache, dizziness, nausea, phonophobia, sleeping difficulties, fatigue, irritable, depressed, frustrated, memory difficulties, concentration difficulties, vision difficulties, photophobia, restlessness | Physio, Specialist Clinic, Up to 8 sessions | Not specified;  Cervical | General physiotherapy; pharmacology; medical care; psychologist; vestibular rehabilitation; oculomotor rehabilitation | NA | RPQ, Active Range of Motion (flexion-rotation test), Goal Attainment Scaling, Torsion Test (SPNTT): To assess smooth pursuit eye movements and neck torsion | No |
| Null and Null, 2019 USA |  | To report on the outcomes of chiropractic care in managing post-traumatic epilepsy and related symptoms and review relevant literature |
| Case Report, N = 1, 19 years old, male, Adult non athlete | Mild,  Long term | Epileptic seizures, dizziness, headache, speech difficulties, mental confusion, neck pain | Chiro, Private clinic, 133 sessions over 2 years | Thrust manipulation;  Cervical | Standalone MT | NA | Self-reported improvement | No |
| Olson et al., 2016 USA |  | To describe the chiropractic management of a student athlete with post-concussion syndrome |
| Case Report, N = 1, 14-year-old male, Teenager athlete | Mild, Subacute | Headache, nausea, vision difficulties, memory difficulties, dizziness, mental confusion, fatigue, irritable | Chiro, Private clinic, 5 sessions over 20 days | Thrust manipulation, soft tissue;  Cervical, thoracic, lumbar | Functional exercises | NA | ImPACT score, Numeric Pain Scale (headache), Self-reported improvement, Romberg test | No |
| Olson et al., 2018 USA |  | To describe chiropractic management and return-to-play of 3 student (American) football players who had experienced concussion |
| Case Report, N = 3, Ages: 16, 15, and 11 years​ (mean age = 14) Gender: Male, Teenager athletes | Mild, Acute and Subacute | Headache, phonophobia, vertigo, concentration difficulties, fatigue, neck pain, dizziness, mental confusion | Chiro, Private clinic, 2 to 3 sessions over 12 to 14 days | Thrust manipulation, soft tissue;  Cervical, thoracic | Education | NA | SCAT 3, return to play, ImPACT score | No |
| Olson et al., 2020 USA |  | To describe the diagnosis and management of a previously unrecognized, sport-related concussion in young adolescent patients. |
| Case Report, N = 1, 10-year-old female, Child | Mild, Subacute | Neck pain, Headache, mid back and low back pain, dizziness, vision difficulties, concentration difficulties | Chiro, Private clinic, 4 sessions over 8 weeks | Thrust manipulation, soft tissue; Cervical, thoracic, lumbar | Education; functional exercises | NA | Child SCAT5, Numeric Pain Scale, ImPACT score | No |
| Patel and Sabini, 2018 USA |  | To determine the safety and potential symptom-relief benefits of OCMM in patients with post-concussion symptoms |
| Prospective observational pilot study, N = 9, Ages: 14–58 years (mean: 27.5 years). Gender: 5 females (55.6%), 4 males (44.4%), Mixed (adult non athletes, teenager athletes and adult athletes) | Mild, Subacute | Headache, dizziness, nausea, phonophobia, sleeping difficulties, fatigue, irritable, depressed, frustrated, memory difficulties, concentration difficulties, vision difficulties, photophobia, restlessness | Osteo, Private clinic, Specialist clinics, 1 session | Functional techniques;  Head | Standalone MT | NA | Adverse events, Post Concussion Symptom Scale (PCSS) | Yes, but none reported |
| Patel, 2020 New Zealand |  | To describe the recovery of post-concussion symptoms and whiplash following chiropractic intervention in a 15-year-old boy |
| Case Report, N = 1, 15-year-old male, Teenager athlete | Mild, Subacute | Neck pain, headache, dizziness, concentration difficulties | Chiro, Private clinic, 10 sessions over 8 weeks | Thrust manipulation, soft tissue, non-thrust manipulation;  Cervical, thoracic | Education; self-management; acupuncture | NA | SCAT 5 | No |
| Remata and Blum, 2021 USA |  | To present a treatment model incorporating sacro-occipital treatment and cranial chiropractic care, along with neurotransmitter balance monitoring, for managing TBI symptoms. |
| Case Report, N = 1, 33-year-old female, Adult non athlete | Mild, Long term | Headache | Chiro, Private clinic, 1 weekly session for 3 years  (156 sessions in total) | Functional techniques;  Head, pelvis | Nutritional supplement | NA | Self-reported improvement | No |
| Reneker et al., 2017 USA |  | To (a) assess the feasibility of recruitment/retention of participants, protocol/resource management, and participant safety, and (b) estimate the size of the effect between the experimental and control groups. |
| RCT, physical therapy = 22 control (sham physical therapy) = 19, Age 10–23, Mixed gender (63.6% male in the experimental group, 57.9% male in control). Mean age: 16.5 years (experimental group), 15.9 years (control group). Mixed (adult athletes and teenager athletes) | Mild, Subacute | Headache, vision difficulties, dizziness, phonophobia, photophobia, nausea, neurological deficit | Physio, Educational institution, Up to 8 sessions over 4 weeks | Soft tissue, non-thrust manipulation, thrust manipulation;  Not specified | General physiotherapy; vestibular rehabilitation; oculomotor rehabilitation; functional exercises | Standardised home exercise program of cervical isometric exercises, range of motion exercises, vestibulo-ocular reflex cancellation exercises | Return to Play, Post Concussion Symptom Scale (PCSS), Adverse events | Yes, but none reported |
| Schneider et al., 2014 Canada |  | To determine if a combination of vestibular rehabilitation and cervical spine physiotherapy reduces time to medical clearance for patients with prolonged post-concussion symptoms |
| RCT, Cervico-vestibular intervention group = 15 Traditional physio control group = 16, Treatment group: Median age = 15, 73.3% male (11/15), 26.7% female (4/15), Control group: Median age = 15, 43.8% male (7/16), 56.3% female (9/16), Mixed (teenager athletes and adult athletes) | Mild, Subacute and Long term | Neck pain, dizziness, headache | Physio, Educational institution, 8 sessions over 8 weeks (1 weekly session over 8 weeks) or until medical clearance | Non thrust manipulation;  cervical, thoracic | General physiotherapy; vestibular rehabilitation | Active range of motion exercises and postural education | Return to Play, Numeric Pain Scale, Activities-specific Balance Confidence Scale, Dizziness Handicap Index, SCAT 2, Head Impulse Test, Vestibular Ocular Motor Screening, Cervical Flexor Endurance, Cervical Joint Position Error Test | No |
| Tomar et al., 2019 India |  | The objective of this study was to observe and compare the effects of manual and mechanical airway clearance techniques on intracranial pressure (ICP) and hemodynamic in patients with severe TBI. |
| RCT, N = 46  cross over design so 23 participants alternating each technique, manual CPT then mechanical CPT = mean age 36.4; 91.3% male (21/23) and 8.7% female (2/23) mechanical CPT then manual CPT = mean age 35; 87.0% male (20/23) and 13.0% female (3/23), Adult non athletes | Severe, Acute | Not applicable | Physio, Hospitals, 1 session of 10 minutes | Non thrust manipulation;  Thoracic | Standalone MT | Mechanical airway clearance using a chest wall vibrator with a frequency of 60 Hz, or 3600 cycles/min | Intracranial pressure (ICP), Cerebral perfusion pressure (CPP), Mean Arterial Pressure (MAP), Heartrate (HR), Arterial Blood Gas (ABG), GCS | Yes, but none reported |
| Wasylynko, 2017 Canada |  | To describe a case of chiropractic care of a patient with non-organic gait disorder that developed after mild head trauma |
| Case Report, N = 1, 10-year-old boy, Child | Mild, Long term | Neurological deficit, neck pain, dizziness, vision difficulties | Chiro, Private clinic, 4 times over a 1-week period and follow-up examination at 3, 6, 1 years later | Thrust manipulation;  Thoracic, lumbar | General physiotherapy; occupational therapy; psychologist | NA | Self-reported improvement | No |
| Wong et al., 2021 USA |  | To describe the clinical and patient reported outcomes of people with post-concussion symptoms following a protocol sequenced to address cervical spine dysfunction and vertigo followed by integrated vision and vestibular therapy. |
| Retrospective longitudinal cohort study, N = 38, Age: 26.9 ± 19.7 years Gender: 25 males, 13 females, Mixed (adult non athletes and adult athletes) | Mild, Subacute and Long term | Neck pain, vision difficulties, dizziness, headache | Physio, Educational institutions; Specialist clinics, 10.4 +/- 4.8 | Not specified;  Cervical | Vestibular rehabilitation; oculomotor rehabilitation; functional exercises | NA | Active Range of Motion, Brock string Test (visual function: measure convergence/divergence distance), Cover uncover test (visual function), Smooth pursuit and saccades, Vestibular measures (dix-hallpike, supine roll tests, vestibular ocular reflex, visual motion sensitivity and balanced Error Scoring System (BESS).), Patient reported outcomes: Post concussion symptom scale (PCSS), Dizziness Handicap inventory, Convergence insufficiency survey (CISS) +  Brain injury vision symptoms survey (BIVSS), Activities-specific Balance Confidence Scale. | No |
| Yao et al., 2020 USA |  | To evaluate the reduction in concussion symptom number and severity through OMM compared to concussion education |
| RCT, Osteopathic Group = 16 Education = 15, Age range: 18-27 years, mean age 19.9 years; Gender: 61.3% male, 38.7% female, Adult Athletes | Mild, Acute and Subacute | Headache, memory difficulties, vision difficulties, nausea, dizziness, sleeping difficulties, photophobia, phonophobia, mental confusion, fatigue, and tinnitus | Osteo, Hospital, 1 session of 30 minutes | Non thrust manipulation, functional techniques;  Head, cervical, thoracic, lumbar | Standalone MT | Education | SCAT 5 | No |
| Yen et al., 2024 Taiwan |  | To evaluate the effects of early progressive mobilization (EPM) compared to early progressive upright positioning (EPUP) on functional outcomes in ICU patients with moderate-to-severe TBI |
| RCT, 65 patients divided in 2 groups: EPM group: 33 or EPUP group: 32, EPM group (Early Progressive Mobilization): Mean age: 53.42 ± 20.52 years Gender: 66.7% male, 33.3% female EPUP group (Early Progressive Upright Positioning): Mean age: 50.78 ± 20.21 years Gender: 65% male, 35% female, Mixed (Adult non athletes and older adults) | Moderate and Severe, Subacute | Neurological deficit | Physio, Hospitals, Daily intervention 5 days a week; 15' to 30' based on tolerance | Non thrust manipulation;  Thoracic, Lower extremity | Pharmacology; medical care | Early progressive mobilisation protocol which consisted of initiating out of bed, task specific activities as soon as possible | Perme ICU Mobility Score (Assesses patient mobility in the ICU, including bed mobility, transfers, gait, and endurance, with higher scores indicating greater independence), Functional Independence Measure (Motor Domain (FIM-Motor) – Evaluates a patient's ability to perform motor tasks related to daily living, such as self-care, transfers, and locomotion), Phase Angle (PhA) from Bioelectrical Impedance Analysis – Measures cell membrane integrity and muscle health, where a higher phase angle indicates better cellular function and nutritional status, Skeletal Muscle Index (SMI) – Assesses muscle mass by calculating lean muscle mass relative to height, helping to evaluate muscle wasting and overall physical condition, ICU length of stay and ventilation duration | Yes,  For both groups, serious adverse events, including falls, unplanned extubating, cardiac arrest, invasive tube dislodgement, or significant arrhythmias, prompted immediate cessation of the intervention. Furthermore, the intervention was temporarily suspended due to ventilator intolerance or intervention intolerance |

Characteristics of included studies

Abbreviations used: 

TBI = Traumatic Brain Injury ; Chiro = Chiropractor; NA = Not Applicable; TMJ = Temporomandibular Joint; RCT = Randomised Controlled Trial; MT = Manual Therapy; Osteo = Osteopath; MET = Muscular Energy Techniques; OMT = Osteopathic Manipulative Treatment; SCAT = Sport Concussion Assessment Tool; Physio = Physiotherapist; ICP = Intracranial Pressure; CPP = Cerebral Perfusion Pressure; PCS = Post Concussion Syndrome; PPCS = Persistent Post Concussion Syndrome; rMSSD = root mean square of successive differences; SLAE = Symptom-Limited Aerobic Exercise; ImPACT = Immediate Post Concussion Assessment and Cognitive Testing; mTBI = mild Traumatic Brain Injury; GCS = Glasgow Coma Scale; RPQ = Rivermead Post Concussion Symptoms Questionnaire; OCMM = Osteopathic Cranial Manipulative Medicine; CPT = Chest Physical; EPM = Early Progressive Mobilization; EPUP =  Early Progressive Upright Positioning ; Therapy; ICU = Intensive Care Unit
